# The Paradox of Progress: A Field Epidemiologist’s Tale of Hepatitis A in Transition

**DOI:** 10.4269/ajtmh.25-0671

**Published:** 2026-03-26

**Authors:** Kunal Pise, Shilpa Tomar

**Affiliations:** Indian Council of Medical Research–National Institute of Virology, Pune, India

I saw his hand trembling as he held her photo and muttered, “She was healthy and then why her?” I could see in his eyes questions that no epidemiological fact sheet could answer. “Why her? And why now?”

Before meeting this father, it was the pumping station that caught my attention during my visit to the small village of Rajur. In the humid April afternoon, I stood there with my team and the local health workers, and I suddenly realized what the villagers went through. The prime water-pumping station had been broken down for over a year. My mind ran the math as my heart sank. Now I understood that I was in the midst of a preventable outbreak of hepatitis A.

About 10,000–15,000 pilgrims visited the local temple at Rajur for a “Jatra” festival, an annual religious gathering that had transformed Rajur. For 3 days, pilgrims gathered to perform the rituals near the Nilwande Dam, which was the main source of drinking water to the villagers of Rajur. The community relied on direct unchlorinated water from the Nilwande Dam. To manage the huge crowd, the organizers provided only eight toilets. This created a terrible ratio of about one toilet for 1,875 pilgrims, which was insufficient. Open defecation near the water source became unavoidable as a result. Untreated water mixed with a massive gathering and poor sanitation followed the typical preventable path for fecal–oral disease spread.

This was an ideal scenario for an outbreak to occur, and so, it did. We saw cases starting to appear 15–20 days after the event ended on March 25, 2025. That timing lined up with the hepatitis A virus incubation period perfectly. The clustering of cases around April 24 and April 28 suggested a common-source exposure.

All of this felt pretty abstract at the start. It just looked like patterns on a spreadsheet and nothing more, but things shifted late one evening in a dimly lit office. Stacks of papers from the outbreak surrounded me everywhere. I was trying to sort through the 0.6% of patients who needed transfer for severe illness. Something caught my attention; one case was of a 13-year-old girl admitted on April 5 and discharged with “fulminant hepatic failure.” The next entry dated 6 days later caught my eye. It was a patient in the same approximate age bracket (20-year-old woman) with an identical diagnosis. Both had been transferred to a tertiary care center. Both had died within 72 hours of admission despite intensive care. Neither patient had any underlying liver disease. The air in that room seemed to thicken around me as I read the final entry of “expired despite [intensive care unit] support” twice. I understood then with a clarity that no statistical summary could convey that these were not data points, but they were two specific, irreplaceable people whose deaths could have been prevented.

We at the ICMR–National Institute of Virology (NIV) in Pune confirmed 327 cases of hepatitis A virus infections of 1,119 suspected ones. The numbers really showed the epidemiology in a straightforward way. The median age turned out to be 15 years old. Eighty-six percent of the cases were in people ages 6–25 years old. That detail stood out as a strong indicator of the hepatitis A transition happening there.

The statistical paradox of hepatitis A infection turning more severe in adolescence and adulthood was not just some data point. It meant two specific tragic deaths for real people. Public health improvements had protected these young women from mild infections during childhood. Yet, those same advances left them vulnerable to deadly disease in their teenage and young adult years. It hit me hard as a clear confirmation. The adolescents in Rajur had paid the price for their community’s partial progress so far.

This ongoing tragedy illustrated how progress could create new vulnerabilities in such a vivid manner. We found a community caught between two worlds. On one side, conditions had improved enough to delay childhood exposure, and on the other side, they were not solid enough to prevent deadly contamination during high-risk events. In classic high-endemic areas, hepatitis A infection remains mild or asymptomatic in kids. It provides lifelong immunity that way. Rajur highlighted the rough edges of incomplete progress clearly. Our analysis uncovered a striking demographic shift that concerned us a lot. Age-specific attack rates reached about 12% in children ages 10–18 years old. Adults older than 30 years old remained protected, with rates less than 1.6%. This pattern confirmed the community’s shift from high to intermediate endemicity.

In the laboratory at the NIV Pune, the molecular data supported the field work as we examined stool samples and identified hepatitis A virus genotype IIIA (a prevalent strain across India). Viral loads hit as high as 10 to the ninth copies, which could easily sustain the outbreak. By combining field epidemiology with molecular sequencing, we could map transmission and distinguish local spread from any imports.

This story extends beyond one village or a district. The state of Kerala experienced 84 hepatitis A outbreaks between 2012 and 2016. Not far away, the Kolhapur district also experiences dozens of outbreaks yearly. Each incident exposes communities suspended between endemic and controlled states. These events arise from failures in India’s water and sanitation systems. Often, they are connected to mass gatherings or other pressures on a fragile infrastructure. The Rajur investigation demonstrated that the real lesson extends beyond immediate causes. It highlights the urgent need for progress across multiple fronts at once, such as improving water treatment infrastructure along with better vaccination strategies and stronger protocols for mass gatherings and surveillance systems.

The father still carries his 13-year-old daughter’s photo with him. The water filtration plant and pump have been repaired by the local authorities. As field epidemiologists, we do more than just identify outbreak sources; we work to ensure that the next community in transition learns from these lessons before tragedy strikes. The paradox of progress is that this advancement that protects children can inadvertently create vulnerability in adolescents. There are miles to go as understanding this paradox is just one step. Acting comprehensively to prevent other adolescent deaths remains the urgent work ahead.

